# iTRAQ-Based Proteomics Analysis of Serum Proteins in Wistar Rats Treated with Sodium Fluoride: Insight into the Potential Mechanism and Candidate Biomarkers of Fluorosis

**DOI:** 10.3390/ijms17101644

**Published:** 2016-09-28

**Authors:** Yan Wei, Beibei Zeng, Hua Zhang, Cheng Chen, Yanli Wu, Nanlan Wang, Yanqiu Wu, Liming Shen

**Affiliations:** 1 Department of Environmental Hygiene, School of Public Health, Guizhou Medical University, Guiyang 550025, China; weiyandc@aliyun.com (Y.W.); 13506904122@163.com (B.Z.); huazhang202@126.com (H.Z.); yangfanwyl@163.com (Y.l.W.); nanlan67@126.com (N.W.); wuyanqiu1257@163.com (Y.q.W.); 2 College of Life Science and Oceanography, Shenzhen University, Shenzhen 518060, China; chenchengszu@163.com; 3 Key Laboratory of Environmental Pollution Monitoring and Disease Control, Ministry of Education, Guizhou Medical University, Guiyang 550025, China

**Keywords:** biomarkers, fluorosis, rats, sodium fluoride, iTRAQ, proteomics, serum

## Abstract

Fluorosis induced by exposure to high level fluoride is quite widespread in the world. The manifestations of fluorosis include dental mottling, bone damage, and impaired malfunction of soft tissues. However, the molecular mechanism of fluorosis has not been clarified until now. To explore the underlying mechanisms of fluorosis and screen out serum biomarkers, we carried out a quantitative proteomics study to identify differentially expressed serum proteins in Wistar rats treated with sodium fluoride (NaF) by using a proteomics approach of isobaric tagging for relative and absolute quantitation (iTRAQ). We fed Wistar rats drinking water that had 50, 150, and 250 mg/L of dissolved NaF for 24 weeks. For the experimental duration, each rat was given an examination of the lower incisors to check for the condition of dental fluorosis (DF). By the end of the treatment, fluoride ion concentration in serum and lower incisors were detected. The results showed that NaF treatment can induce rat fluorosis. By iTRAQ analysis, a total of 37 differentially expressed serum proteins were identified between NaF-treated and control rats. These proteins were further analyzed by bioinformatics, out of which two proteins were validated by enzyme-linked immunoadsorbent assays (ELISA). The major proteins were involved in complement and coagulation cascade, inflammatory response, complement activation, defense response, and wound response, suggesting that inflammation and immune reactions may play a key role in fluorosis pathogenesis. These proteins may contribute to the understanding of the mechanism of fluoride toxicity, and may serve as potential biomarkers for fluorosis.

## 1. Introduction

Fluorine is a common element in the earth’s crust, and it is a highly reactive element that can be combined with other elements to form fluorides [1]. Fluoride is abundant and exists in the soil, rocks, and water. Humans may intake excessive fluoride from water, food, and air, which can develop into dental, skeletal, and non-skeletal fluorosis. Because of the high chemical and biological activity, fluoride can migrate freely and rapidly across biological membranes and bind with biomacromolecules, and therefore induce cell damage. Metabolic, functional, and structural damage caused by chronic fluorosis have been reported in many tissues. The manifestations of fluorosis include dental mottling, bone damage, and impaired malfunction of soft tissues, including the liver, thyroid, kidney, gonad, and nervous system [2,3,4,5,6,7,8,9,10,11]. Fluoride dose-effects on the different organs given different types of fluorosis, for example, 1.5 ppm fluoride in drinking water is associated with dental and skeletal fluorosis, and signs of skeletal fluorosis become evident on consumption of 8–10 ppm of fluoride in drinking water for approximately 10 years or more [12].

Dental damage and skeletal damage are both the most common manifestations of fluoride toxicity. Dental fluorosis is a disturbance in tooth formation caused by the excessive intake of fluoride during the formative period of dentition, and results in the mottling of dental polish, hard and fragile [13,14]. The most severe manifestation of fluorosis is bone damage which features osteosclerosis, ligament calcifications, and often osteoporosis, osteomalacia, or osteopenia [15,16]. Patients suffering from skeletal fluorosis are always with significant long-term difficulties, including impaired neck and lumbar mobility, aching of the axial skeleton, kyphosis, and painful lower extremities, ultimately causing crippling and incapacitation [17,18,19]. The existing evidence has shown that fluoride effects in adults and in early infancy are different; for example, the main sign in children is dental damage [20]. Dental fluorosis is the first sign of excessive fluoride intake. In adults, tooth development is finished and excessive fluoride leads to bone fluorosis [20,21]. Fluorosis induced by exposure to high fluoride in the environment is quite widespread in many countries. In China, the problem of high fluoride concentration in groundwater, coal-burning food, and drinking brick tea is one of the important toxicological and geo-environmental issues. It was estimated that the prevalence of endemic fluorosis affects more than 100 million people and 2.9 million people suffer from skeletal fluorosis in China [22].

To date, the molecular mechanism of skeletal fluorosis has yet to be further studied, and the diagnostic biomarker related to the disease mechanism is absent. The proteomics analysis associated with fluoride has been studied in different tissues [10,23,24], but not in serum. Serum proteomics is a very attractive approach to disease biomarker discovery because testing of biomarkers in blood is simple, safe, and minimally invasive [25]. Differentially expressed proteins in serum could also provide some information to elucidate the molecular mechanisms of fluorosis. Among the proteomics techniques, iTRAQ (isobaric tags for relative and absolute quantitation) has the following advantages: allowing simultaneous comparison of protein profiles in several samples; being a relatively high throughput process; and providing information on peptide quantitation and identification [26,27,28]. A typical workflow of iTRAQ analysis includes the extraction of proteins from tissues/cells/blood, digestion with trypsin, labeling of peptides with iTRAQ reagents, fractionation of labeled peptides and finally the analysis of the sample by using high performance liquid chromatography–tandem mass spectrometry. NaF is one of the most common inorganic fluorides, which is frequently used in the research of fluoride toxicity. NaF induced cell damage has been demonstrated in different organs and tissues. Here, we present the first report of the differential expression of serum proteins in NaF-treated rats compared with the normal controls by the iTRAQ method. Our results suggest that identified proteins are associated with inflammation and immune reaction, which is likely to elucidate better understanding of fluorosis pathobiology and serve as candidate markers for its diagnosis.

## 2. Results

### 2.1. Analytical Strategy for Rat Serum Proteome Identification under NaF Treatment

**Figure 1:**
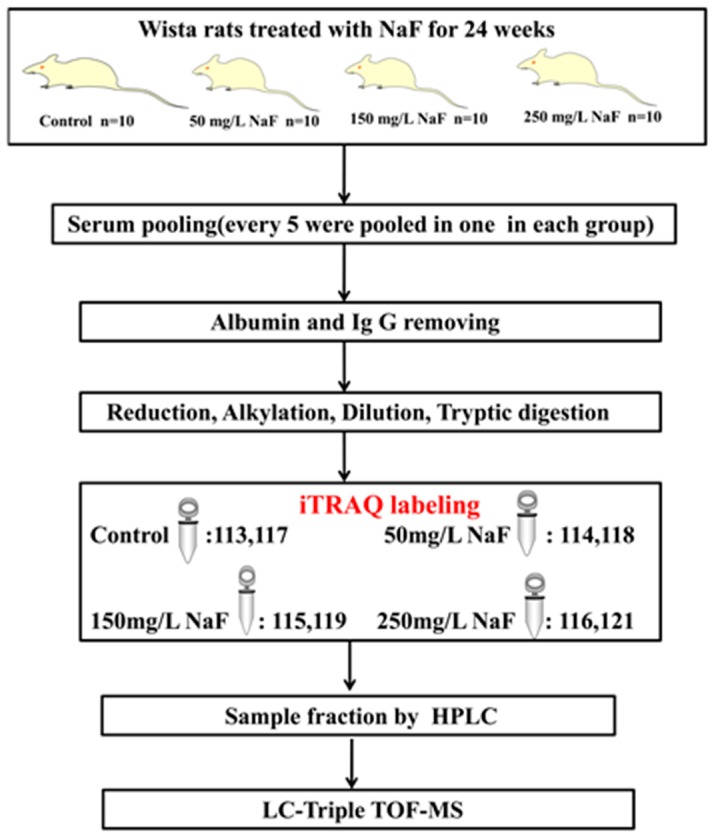
General workflow of the iTRAQ experiment of the rats with fluorosis. Rats were divided randomly into four groups: Control group, 50 mg/L NaF group, 150 mg/L NaF group, 250 mg/L NaF group. NaF treatment lasted for 24 weeks. An equal volume of serum from each rat was pooled, and every five rats’ serum in the same group was pooled in one sample. An iTRAQ-based quantitative proteomics analysis for serum was used to gain a global view of proteome responses to different doses of NaF treatments. 114/113 and 118/117 were considered as the two replicates for the 50 mg/L group, 115/113 and 119/117 were considered for the 150 mg/L group, and 116/113 and 121/117 were considered for 250 mg/L group.

The analytical strategy of the iTRAQ-based quantitative proteomics approach used in the study is shown in Figure 1.

### 2.2. NaF Induced Dental Fluorosis in Wistar Rats

**Figure 2:**
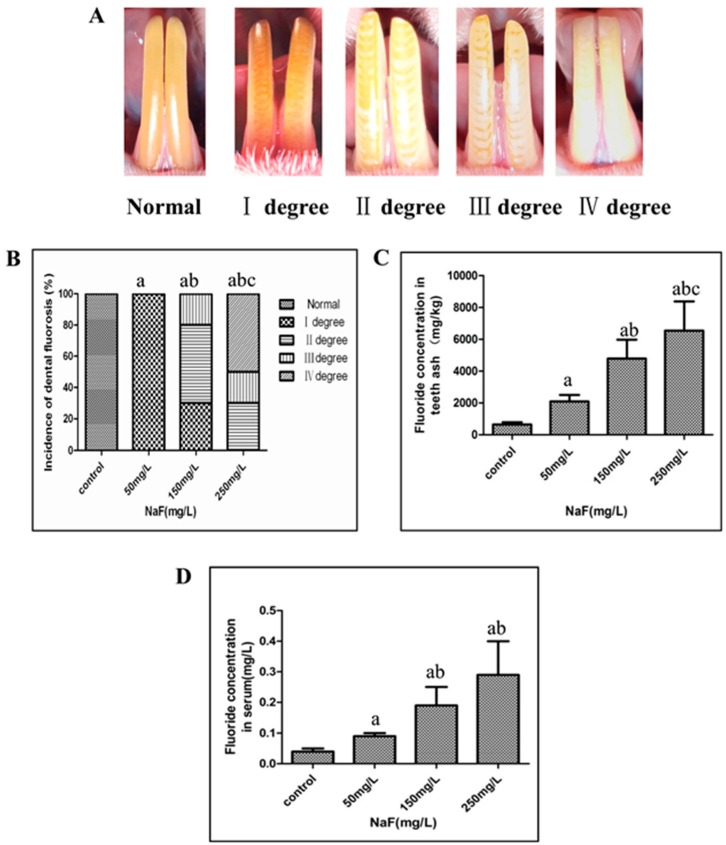
NaF induced fluorosis in Wistar rats. (**A**) Representative dental fluorosis (DF) photos of each degree; (**B**) Comparison of DF severity distribution among control, 50, 150, and 250 mg/L groups. Data plotted as bars are the incidence rates (%), and the Kruskal-Wallis H rank sum test was used to analyze the difference of DF severity distribution among the four groups. The letter “a” indicates *p* < 0.05 vs. control group, “b” is *p* < 0.05 vs. 50 mg/L group, and “c” is *p* < 0.05 vs. 150 mg/L group; (**C**,**D**) Comparison of fluoride concentration in lower incisor ash and serum among control, 50, 150, and 250 mg/L groups. Data are plotted as mean ± SD of ten rats in each group. The letter “a” indicates *p* < 0.05 vs. control group, “b” is *p* < 0.05 vs. 50 mg/L group, and “c” is *p* < 0.05 vs. 150 mg/L group.

NaF treatment for 24 weeks can induce fluorosis in Wistar rats. Rats in the 250 mg/L group had the most severe toxicity condition. When the dose of NaF was lower, the rats’ toxicity condition was milder (Figure 2).

Normal rats had the deep yellow and translucent tooth surface. When NaF intake increased, the white area of the tooth surface became wider, and more serious defects can also be observed. The severity of dental fluorosis (DF) was classified into four degrees and the representative photo of each degree is shown in Figure 2A. By the end of NaF treatment, no DF rat was observed in the control group, however, the DF incidence rate of each experimental group was 100%. In detail, incidence of I degree DF was 100% in the 50 mg/L group; I, II, III degree DF was 30%, 50%, and 20% in the 150 mg/L group, respectively; II, III, IV degree DF was 30%, 50%, and 20% in the 250 mg/L group respectively, and statistical differences of the severity distribution among the four groups were present (Figure 2B).

To confirm whether the internal dose agreed with the external dose and DF severity, the fluoride concentration in serum and tooth was analyzed by means of a combination fluoride-specific electrode and pH/ion meter. As expected, fluoride concentrations of the three experimental groups were higher than those of the control group in both samples. When the external concentration increased, the internal concentration also became higher (Figure 2C,D).

### 2.3. Serum Protein Expression Profiles of NaF Treated Rats

A total of 432 non-redundant proteins were identified (Table S1). The number of identified proteins with an unused score >1.3 was 336. Among the 336 proteins, the number of peptides ≥2 was 266. Proteins with a *p* value <0.05 and more than 1.2-fold changes were considered as the differentially expressed protein. As shown in Table 1, a total of 37 proteins were identified. Among them, in the 50 mg/L group, the number of differentially expressed proteins was 18 (10 proteins were up-regulated and 8 were down-regulated). In 150 and 250 mg/L group, the number was 21 (6 proteins were up-regulated and 15 were down-regulated) and 15 (6 proteins were up-regulated and 9 were down-regulated), respectively. Three uncharacterized proteins were identified as the fragments of protein Ighm.

**Figure 3:**
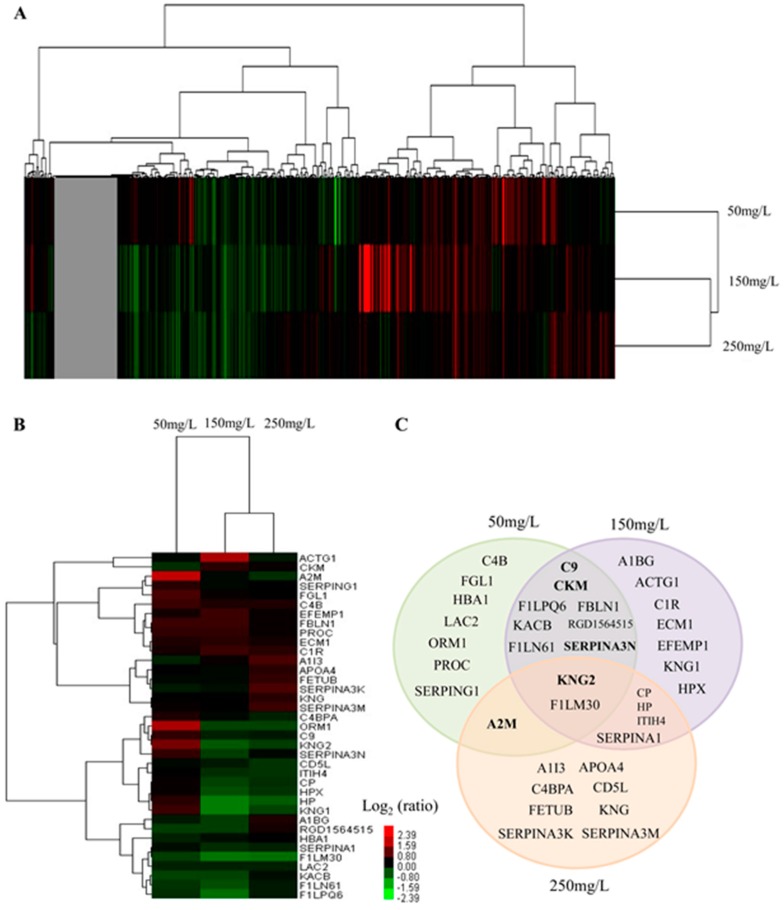
An overview of serum protein expression patterns between NaF-treated and control rats. (**A**) Cluster map comparing the protein expression patterns of different NaF-treated groups. Red indicates higher expression, green indicates lower expression, and black indicates the same expression levels compared with the control; (**B**) Cluster map comparing the differentially expressed proteins identified in different NaF-treated groups; (**C**) Venn diagram depicted the overlapping of differentially expressed proteins identified in different treatment groups. The proteins that displayed different variation trends between different treatment groups are indicated in bold.

The 266 proteins and 37 differentially expressed proteins were further analyzed by cluster analysis using the Cluster 3.0 software (Michael Eisen, Stanford University, Stanford, CA, USA). Interestingly, we observed a homology not only in the expression pattern of proteins but also in the total identified proteins in both the study groups i.e., 150 and 250 mg/L groups as shown in (Figure 3A,B). As shown in Figure 3B, expression levels of A2M, C4BPA, ORM1, C9, KNG2, SERPINA3N, CP, HPX, HP, and KNG1 showed an increasing trend in the 50 mg/L group, and in contrast decreasing trend in the 150 and/or the 250 mg/L group. Five proteins (A1BG, RGD1564515, F1LN61, F1LM30, and F1LPQ6) revealed a decreasing trend in the 50 and 150 mg/L groups. The expression level of F1LM30 was also decreased in the 250 mg/L group.

The overlapping of differentially expressed proteins in different treatment groups was illustrated by Venn diagram analysis and is shown in Figure 3C. Among the 37 proteins, KNG2 and F1LM30 were identified as differentially expressed proteins in all three groups. There are 7, 7, and 8 proteins identified as differentially expressed proteins only in the 50, 150, and 250 mg/L groups, respectively. There were 8, 4, and 1 proteins that were found to be common between the 50 and 150 mg/L groups, between the 150 and 250 mg/L groups, and between the 50 and 250 mg/L groups, respectively. However, as shown in Table 1 and Figure 3C, some proteins (indicated in bold in Figure 3C) displayed different variation trends between different treatment groups.

### 2.4. Functional Classification and Protein-Protein Interaction Analysis of Differential Expressed Proteins

**Figure 4:**
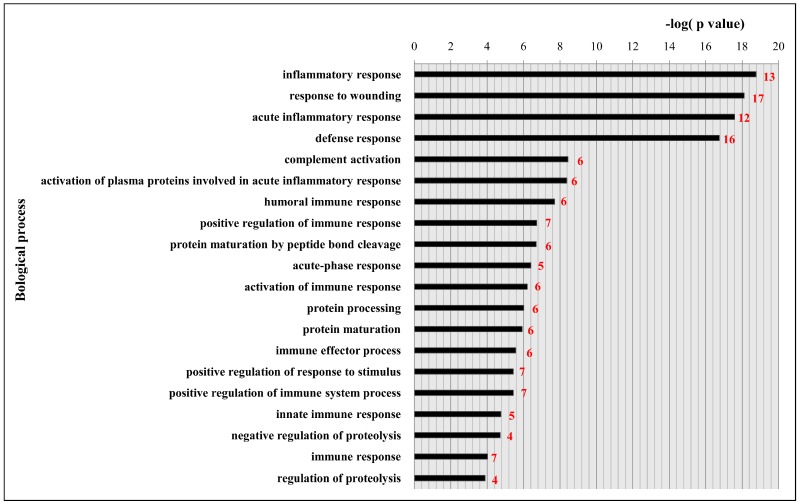
Gene ontologies enriched amongst all differentially expressed proteins. The top 20 enriched biological process. The enrichment analysis was performed using the DAVID (Database for Annotation, Visualization and Integrated Discovery) online bioinformatics software. The number of proteins in each category is shown beside the bar (i.e., red number).

The DAVID (Database for Annotation, Visualization and Integrated Discovery) database was used to elucidate the biological processes of differentially expressed proteins, and the ranking of the top 20 biological process is shown in Figure 4. These proteins were involved in the following processes: inflammatory response (46.9%), response to wounding (53.1%), acute inflammatory response (37.5%), defense response (50.0%), complement activation (18.8%), activation of plasma proteins involved in acute inflammatory response (18.8%), humoral immune response (18.8%), positive regulation of immune response (21.9%), protein maturation by peptide bond cleavage (18.8%), acute-phase response (15.6%), activation of immune response (18.8%), acute-phase response (15.6%), activation of immune response (18.8%), protein processing (18.8%), protein maturation (18.8%), immune effector process (18.8%), positive regulation of response to stimulus (21.9%), positive regulation of immune system process (21.9%), innate immune response (15.6%), negative regulation of proteolysis (12.5%), immune response (21.9%), and regulation of proteolysis (12.5%). Clearly, most proteins were related to the inflammation and immune reaction, response to wounding, defense response, and complement activation.

**Figure 5:**
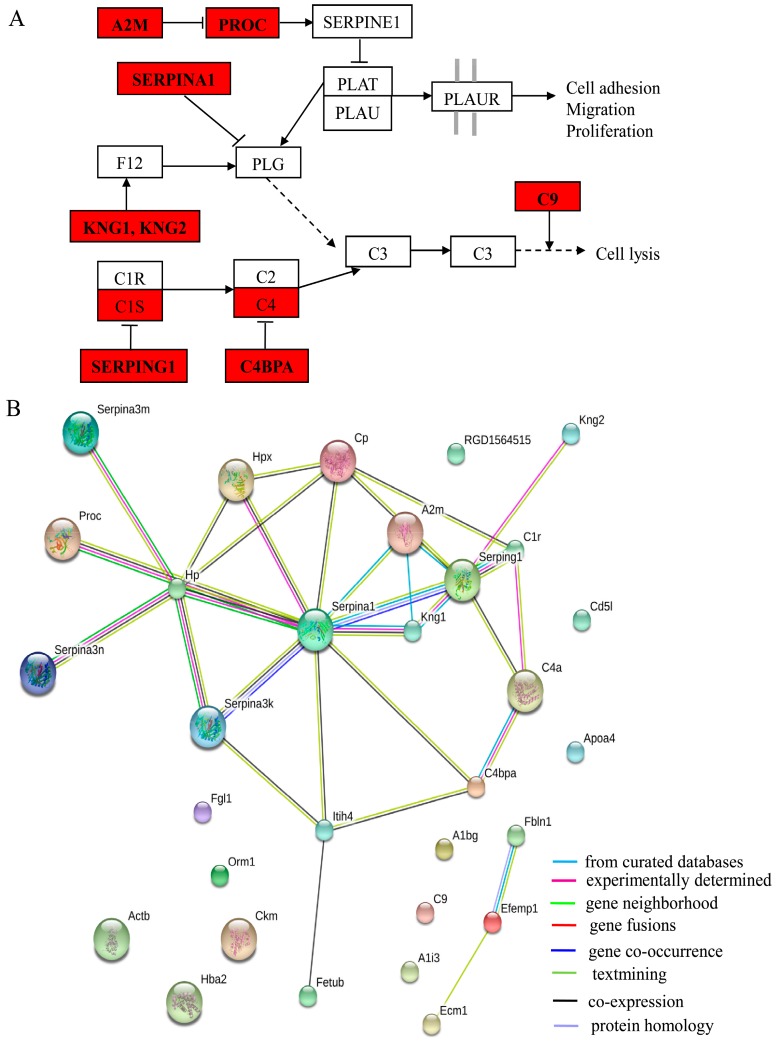
Functional networks of identified proteins. (**A**) Pathway analysis using the DAVID website database. (KEGG ID: rno04610, Complement and coagulation cascades). Proteins with red shading were differentially expressed; (**B**) Protein network analysis of the differentially expressed proteins using the online tool STRING v9.1. The color of the connective lines indicates the type of evidence for the connection.

By pathway analysis, 10 proteins were involved in the KEGG (Kyoto Encyclopedia of Genes and Genomes) pathway of complement and coagulation cascade (*p* = 9.5 × 10^−16^). The 10 proteins were A2M, C1R, C4B, C4BPA, C9, KNG1, KNG2, PROC, SERPING1, and SERPINA1. The relationships between them in the pathway are shown in Figure 5A. In addition, the protein-protein interaction (PPI) networks associated with differentially expressed proteins were generated by the STRING database and are shown in Figure 5B. Except for C9, the proteins involved in this pathway are tightly networked.

### 2.5. Validation of Differential Expression of Proteins Found in iTRAQ via ELISA Assay

**Figure 6:**
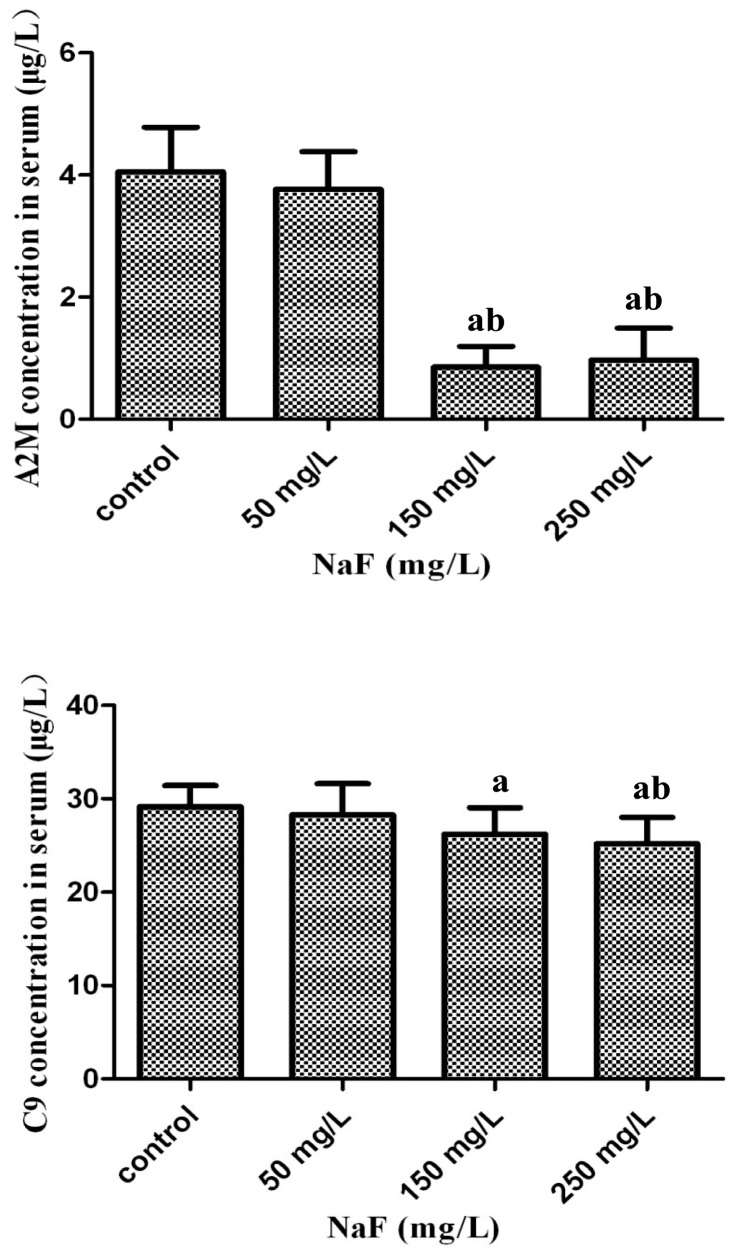
Validation of differentially expressed proteins in the rat serum with fluorosis by ELISA. Data are mean ± SD of ten rats in each group. The letter “a” indicates *p* < 0.05 vs. control group, “b” indicates *p* < 0.05 vs. 50 mg/L group.

As mentioned above, inflammation and immune reaction proteins may be involved in the pathogenesis of fluorosis. Thus, we chose these kinds of proteins, A2M and C9, to confirm this by ELISA analysis. They exhibited differential expression in iTRAQ analysis in at least two groups and we obtained their commercial ELISA kits. As shown in Figure 6, the results were partially consistent with the iTRAQ analysis, and the proteins were not found to be significantly increased in the 50 mg/L group. However, consistent with the proteomics results, the expression levels of both the proteins were decreased significantly in the 150 and 250 mg/L groups (*p* < 0.05). Thus, the expression levels of these two proteins in the 50 mg/L group need to be further validated.

## 3. Discussion

In this study, to search for biomarkers and clarify the molecular changes that occurred in Wistar rat serum, we fed rats with drinking water containing different concentrations of NaF. As expected, DF diagnosis and fluoride concentration in serum and tooth determination indicated that NaF treatment can induce rat fluorosis, and the rats’ toxicity state matched the dose of NaF. Subsequently, we identified and quantified differentially expressed serum proteins in rats with fluorosis compared with controls. This is the first report that applies iTRAQ proteomics techniques to the serum of animals with fluorosis.

An expression of 37 proteins was found altered during this study. By using some bioinformatics tools, it was assured that 10 proteins were involved in KEGG pathways related to complement and coagulation cascade systems. These systems are closely linked and perform the function of homeostasis. Among the two, the complement functions against pathogens invading the body as being part of the innate system of immunity. It performs in following three ways, mobility of the immune cells at the infection site, opsonization for proper identification of the pathogens to ensure the proper intake and lyses by phagocytes and the at the end, the direct destruction of the vulnerable pathogens. More than 35 proteins directly participate in complement activities. Some of them include circulating zymogens, membrane bound regulators and cell bound receptors. The pathways recognized for complement activation include the lectin pathway, classical pathway, and the alternative complement pathway [29,30,31]. These pathways ultimately result in protein cleavage and lysis of C3 resulting in assemblage of C6, C7, C8, and C9 terminal complement components into the membrane attack complex (MAC). This complex destroys the target cells by forming certain channels in the target membranes, hence, disrupting their proper function [32,33]. Interestingly, the proteins we identified in the pathway of complement and coagulation cascade can affect C3 indirectly in a different way as explained in (Figure 5A).

Likewise, the coagulation system plays a role in fighting infections [34] and is implicated in the pathophysiology of several diseases along with its role in the maintenance of hemostasis. Both systems can be viewed as partners in an inflammation that is aimed at stabilizing a living system that has encountered various disturbances to its homeostasis [35]. In the present study, GO (Gene Ontology) showed that the top five biological processes that the differentially expressed proteins involved in were inflammatory response, response to wounding, acute inflammatory response, defense response, and complement activation, suggesting that inflammation and immune reaction may play a key role in fluorosis pathogenesis.

Inflammation is the first response of the immune system to infection or tissue damage. Chronic inflammation is harmful and may play an important role in the development of many diseases such as diabetes, osteoarthritis, and rheumatoid arthritis. Fluoride exposure has been implicated in inflammation. Human exposure to hydrogen fluoride induced an immediate nasal inflammatory reaction, in which an increase of tumor necrosis factor-α (TNF-α) was observed [36]. Wistar rats in the second generation of adult rats exposed to 50 or 100 mg/L NaF via drinking water were observed to have inflammatory infiltrations in peribromchial, perivascular, intraparenchymal, and respiratory tract lumen [37]. The inflammatory effect of fluoride exposure was also evaluated in human lung epithelial cells, and the activity of IL-8 was found to be increased 5- to 7-fold after 20 h exposure to 3.75 and 5 mM NaF, respectively [38]. Thus, all of these findings agree with our results.

A2M is a protease inhibitor and carrier for several growth factors and cytokines, including TNF-α, IL-1β, IL-6, and TGF-β [39]. It is considered an acute protein and has been found to be associated with many diseases. Protein C is a vitamin K-dependent, serine protease that is found in the blood plasma and synthesized in the liver. The active form of protein C can inhibit blood coagulation and stimulate fibrinolysis [40]. SERPING1 plays an important role in the complement pathway, by inhibiting the activation of the classical and lectin complement pathways by suppressing the activity of complement component 1 and mannan-binding lectin serine peptidase 2 [41,42,43]. SERPINA1 is the most abundant proteinase inhibitor in the circulation and extracellular fluids, and is also an irreversible inhibitor of neutrophil elastase and other serine granule peptidases, such as proteinase 3 and cathepsin G [44,45,46,47]. KNGs are plasma glycoproteins that were first identified as acute phase reactants in rat serum and later found to be cysteine proteinase inhibitors. There are four types of KNGs in rats, two of which are classical high and low molecular weight KNGs, and the other two are low molecular weight-like KNGs designated as KNG1 and KNG2. Both KNG1 and KNG2 are 430 amino acid secreted rat proteins that each contain three cystatin domains and have nearly identical functions [48]. C4b-binding protein (C4BP) is the most important soluble regulator of the classical pathway of complement activation [49]. In humans, C4BP is a hetero-oligomer assembled by two types of polypeptide chains: seven α-chains (C4BPA) and one β-chain (C4BPB) [50,51]. C4BPA bears the complement regulatory function of C4BP by binding and inactivating the C4b component of the classical pathway C3-converse [50]. As mentioned above, MAC is a sequential self-assembly of C6, C7, C8, and C9. C9 is a single-chain protein that has the unique ability to polymerize during MAC formation [52,53]. Taken together, our results suggest that inflammation may be involved in the pathogenesis of fluorosis, and we report for the first time those 13 proteins that may play a role in fluorosis via the inflammation and immune pathways.

It is worth noting that among the 10 proteins associated with complement and coagulation cascade, there are 5 proteins (A2M, C4BPA, C9, KNG1, and KNG2) that were down-regulated in the moderate- and/or high-dose group (150 and 250 mg/L groups), whereas an increasing trend was exhibited in the low-dose group (50 mg/L group). Similarly, among the 13 proteins associated with inflammation and immune reactions, there are 8 proteins (ORM1, A2M, C4BPA, C9, HP, SERPINA3N, KNG1, and KNG2) that also display the same trend. Taken together with the expression pattern of the total proteins, this implies that low-dose NaF may promote complement, inflammation, and immune responses, whereas moderate- and high-dose NaF may inhibit these responses. Further study will be focused on verifying the relationship between these proteins and the pathogenesis of fluorosis.

In addition, the differentially expressed proteins identified in this study may serve as candidate biomarkers for fluorosis. Since some proteins were independently altered in a treatment group and other proteins were altered in two or three treatment groups, this suggests that these proteins could serve as potential protein biomarkers for the monitoring of disease degree and course. Further studies are needed to confirm these results. Of note, fluoride effects on bone are of particular interest and we will carry out further proteomics analyses of bone tissue, and investigate the relationship of the different expression of proteins between the serum and bone tissue. Moreover, these proteins relate to the cause of the fluorosis pathology or are a consequence of the pathology, and this mechanism need to be further clarified.

## 4. Experimental Section

### 4.1. Animals and Treatment

Wistar-strain rats (weighing between 80–120 g) were obtained from Experimental Animal Center of Guizhou Medical University and were kept in ventilated cages at 25–28 °C and 12/12 h light-dark cycles with free access to food and water. All procedures were carried out according to the guidelines of the Animal Care Welfare Committee of Guizhou Medical University (approval number: 201312024, 24 December 2013). Rats were divided randomly into four groups containing 10 animals per group (an equal number of males and females were included): (1) Control group: received tap water. Chemical analysis showed fluoride ion concentration was less than 0.50 mg/L, which was within the normal limits; (2) 50 mg/L NaF group: received tap water supplemented with 50 mg/L NaF (assay ≥99%, No.: 7681-49-4, Product No.: S1504; Sigma Chemical Co., St. Louis, MO, USA), and the fluoride ion concentration was 22.62 mg/L; (3) 150 mg/L NaF group: received tap water supplemented with 150 mg/L NaF and the fluoride ion concentration was 67.86 mg/L; (4) 250 mg/L NaF group: received tap water supplemented with 250 mg/L NaF and the fluoride ion concentration was 113.11 mg/L. The dose design was based on LD50 of NaF [54], as well as the daily water consumption of rats [55].

NaF treatment lasted for 24 weeks. Every two weeks, each rat was given an examination of the lower incisors to check for the condition of dental fluorosis. By the end of the treatment, rats were anesthetized by ether inhalation and were then killed. Serum and lower incisors were collected for fluoride ion detection and serum was collected for proteomics analysis.

### 4.2. Classification Principle for Rat’s Dental Fluorosis (DF)

Rats’ dental fluorosis was judged with the following standard: (1) Normal: deep yellow, translucent tooth surface; (2) I degree: Fine, regular, yellow, and white cross striation can be observed on the tooth surface (the width of the striation is not more than about 1 mm); (3) II degree: Yellow and white cross striation becomes wide (more than 1 mm) and irregular, but the white area is less than 50% of the whole surface; (4) III degree: White area of the yellow and white cross striation is more than 50% of the whole surface; (5) IV degree: Along with the wide and irregular cross striation, defects are also observed on the tooth surface or edge.

### 4.3. Fluoride Ion Concentration Analysis

The rat lower incisor was placed in a porcelain crucible. After ashing at 650 °C for 2 h, all of the tooth ash was weighed and dissolved in 2–3 mL of 3 mol/L HCL, and then 2 mol/L NaOH was used to adjust the pH value to 5–6. Subsequently, fluoride ion concentration was analyzed by means of a combination fluoride-specific electrode (Model pF-1-01, INESA Scientific Instrument Co., Ltd., Shanghai, China) and pH/ion meter (Model PHS-3C, INESA Scientific Instrument Co., Ltd.). Serum samples were analyzed directly for fluoride ion concentration by the same method.

### 4.4. Depletion of High-Abundance Proteins

An equal volume of serum from each rat was pooled, and every five rats’ serum in the same group was pooled into one sample. The pooled samples were treated with ProteoExtract Albumin/IgG Removal Kit (Calbiochem, Darmstadt, Germany) to remove albumin and immunoglobulin (IgG), according to the manufacturer′s instructions. Then the samples were centrifuged in a YM-3 centrifugal filter (Millipore, Darmstadt, Germany) at 12,000× *g* and were then buffer-exchanged with sample buffer (7 M urea, 2 M thiourea, 4% CHAPS, 65 mM DTT, 30 mM Tris). Serum proteins concentrations were determined using the Bradford assay. All protein samples were stored at −80 °C until further analysis was performed.

### 4.5. Protein Digestion and iTRAQ Labeling

Immunodepleted serum protein (100 µg) was reduced and alkylated by 10 mM DTT (dithiothreitol, Sigma-Aldrich Co., St. Louis, MO, USA) at 37 °C for 1 h and 50 mM iodoacetamide (IAA, Sigma-Aldrich) in the dark at room temperature for 10 min. The samples were desalted and buffer-exchanged three times with 100 μL 0.5 M triethylammonium bicarbonate (TEAB, AB Sciex, Foster City, CA, USA) with Ultra Centrifugal Filters (Amiconl Ultra-15, Millipore). After that, proteins were digested using sequencing grade trypsin (Promega, Madison, WI, USA) at a ratio of 1:20 (*w*:*w*) at 37 °C overnight. Finally, digested peptides were labeled with the iTRAQ reagents according to the protocol of the 8-plex iTRAQ labeling kit (AB Sciex). The two samples from the control group were labeled with iTRAQ tags 113 and 117, the 50 mg/L NaF group with 114 and 118, the 150 mg/L NaF group with 115 and 119,and the 250 mg/L NaF group with 116 and 121, respectively.

After labeling, the samples were incubated at room temperature for 1 h, and then the labeled samples were mixed and lyophilized. The dried samples were reconstituted in 100 μL deionized water and injected into an Agilent high-performance liquid chromatography (HPLC) system (Agilent Technologies, Santa Clara, CA, USA) with a high pH reverse phase (RP) column (Durashell, C18, 250 mm × 4.6 mm, 5 μm; Bonna-Agela Technologies Inc., Wilmington, DE, USA). A total of 48 fractions were collected, merged into ten pooled fractions, and lyophilized. Prior to LC-MS/MS analysis, the ten pooled fractions were reconstituted in 30 μL of LC-MS buffer and centrifuged at 12,000× *g* for 10 min, with 8 μL of each fraction used per nanoLC-MS/MS analysis.

### 4.6. LC-MS Analysis

Peptide separation was performed on an ekspert ultra LC 100-XL (Eksigent Inc., Dublin, CA, USA) equipped with a Triple TOF 5600 system (AB Sciex). Microfluidic traps and nanofluidic columns packed with ChromXP C18 (3 μm, 2.1 mm × 100 mm, Eksigent) were utilized for online trapping and desalting, while nanofluidic columns packed with ChromXP C18 (3 μm × 150 cm, Eksigent) were employed in analytical separation. The mass spectrometry data was acquired in the positive ion mode, with a selected mass range of 350–1500 *m*/*z*. Peptides with +2 to +5 charge states were selected for MS/MS. MS/MS spectra were acquired in the mass range of 100–1500 *m*/*z*. Smart information-dependent acquisition (IDA) was activated with automatic collision energy and automatic MS/MS accumulation.

### 4.7. Database Search and iTRAQ Data Analysis

Protein identification and quantification were performed using ProteinPilot v4.0 (AB Sciex) with the Paragon Algorithm against the rat Swiss-Prot database. The parameter sets were as follows: maximum missed trypsin cleavages = 2; peptide mass tolerance = 25 ppm; MS/MS tolerance = 0.2–0.3 Da; iTRAQ labeling of lysine, peptide with N termini, and cysteines by methyl methanethiosulfonate were specified as fixed modification.

Peak areas of each peptide at *m*/*z* 113–119 and 121 were used for protein quantification. Samples labeled with 113 and 117 were used as a reference. Average peak area ratios, 114/113, 115/113, 116/113, 118/117, 119/117, and 121/127 were calculated for differential protein identification. 114/113 and 118/117 were considered as the two replicate for the 50 mg/L group. Similarly, 115/113 and 119/117 were considered for 150 mg/L group, and 116/113 and 121/117 were considered for 250 mg/L group, respectively. To be identified as being significantly differentially expressed, a protein must have had at minimum 2 unique peptide matches and a *p*-value < 0.05, and the ratio change must have been more than 1.2 or less than 0.8 in both replicates.

### 4.8. Bioinformatics Analysis

The differentially expressed proteins were entered into the DAVID database for functional analysis. Protein-protein interaction (PPI) networks associated with these proteins were analyzed with the STRING database (Search Tool for the Retrieval of Interacting Genes/Proteins, Version 9.1) at the website: http://string-db.org/.

### 4.9. ELISA Analysis for Protein Validation

To confirm the iTRAQ proteomics results, levels of some proteins in each rat serum were measured using a mouse ELISA Kit (BioTsz, San Francisco, CA, USA), according to the manufacturer′s instruction.

### 4.10. Statistical Analysis

Fluoride concentration of serum and teeth ash and ELISA results of serum protein is shown as mean ± SD. One-way ANOVA followed by the Student-Newman Keuls (SNK) method was used to test the difference. Difference of dental fluorosis incidence of each group was analyzed with Kruskal-Wallis H rank sum test. Statistical analysis was performed with SPSS software (SPSS Inc., Chicago, IL, USA). Statistically significant level was considered as α = 0.05.

## Figures and Tables

**Table 1:** Summary of the proteins identified as differentially expressed using the iTRAQ-based quantitative proteomics approach.

Protein Name	Gene Name	Swissport Accession No.	Protein Score	Coverage (%)	No. of Peptides	50 mg/L NaF vs. Control (Ratio)	150 mg/L NaF vs. Control (Ratio)	250 mg/L NaF vs. Control (Ratio)
α-1-inhibitor 3	A1I3	P14046	135.27	73.3	107	0.80	1.11	**1.52** (↑) *
Actin, cytoplasmic 2	ACTG1	P63259	18.87	52.8	11	0.92	**2.60** (↑) *	0.84
α-1-acid glycoprotein	ORM1	P02764	11.39	39.5	8	**2.60** (↑) *	0.65	0.64
α-1-antiproteinase	SERPINA1	P17475	40.96	61.3	33	0.85	**0.73** (↓) *	**0.78** (↓) *
α-1B-glycoprotein	A1BG	Q9EPH1	36.84	52.2	27	0.81	**0.66** (↓) *	1.24
α-2-macroglobulin	A2M	P06238	67.4	43.7	43	**3.01** (↑) *	0.90	**0.73** (↓) *
Apolipoprotein A-IV	APOA4	P02651	46.78	83.9	40	0.99	0.93	**1.28** (↑) *
C4b-binding protein α chain	C4BPA	Q5M891	18.71	28.3	10	1.23	0.9	**0.80** (↓) *
C9 protein	C9	Q5BKC4	41	55.2	29	**1.34** (↑) *	**0.78** (↓) *	0.82
CD5 antigen-like	CD5L	Q4KM75	20	42.8	10	0.93	0.83	**0.71** (↓) *
Ceruloplasmin	CP	G3V7K3	84.04	63.3	57	1.07	**0.65** (↓) *	**0.73** (↓) *
Complement component 4, gene 2	C4B	Q6MG90	15.46	40	46	**1.45** (↑) *	1.18	1.23
Creatine kinase M-type	CKM	P00564	11.05	31.5	6	**0.73** (↓) *	**1.30** (↑) *	1.11
EGF-containing fibulin-like Extracellular matrix protein 1	EFEMP1	Q6AXN2	5.56	22.1	3	1.17	**1.42** (↑) *	1.07
Extracellular matrix protein 1	ECM1	Q62894	12	33.6	6	1.19	**1.30** (↑) *	1.17
Fetub protein	FETUB	Q6IRS6	33.22	66.7	22	0.93	0.92	**1.34** (↑) *
Fibrinogen-like protein 1	FGL1	Q5M8C6	3.51	17.8	3	**1.50** (↑) *	1.08	1.02
Fibulin 1 (Predicted)	FBLN1	D3ZQ25	10.57	30.2	6	**1.40** (↑) *	**1.40** (↑) *	1.09
Haptoglobin	HP	P06866	32.67	63.7	24	1.26	**0.44** (↓) *	**0.55** (↓) *
Hemoglobin subunit α-1/2	HBA1	P01946	11.09	57	8	**0.78** (↓) *	0.89	0.95
Hemopexin	HPX	P20059	52.31	68.7	37	1.09	**0.60** (↓) *	0.72
Igκ chain C region, B allele	KACB	P01835	16.1	95.3	15	**0.65** (↓) *	**0.63** (↓) *	0.86
Igλ-2 chain C region	LAC2	P20767	12	87.5	10	**0.77** (↓) *	0.77	0.79
Inter α-trypsin inhibitor, heavy chain 4	ITIH4	Q5EBC0	72.55	60.1	46	1.00	**0.75** (↓) *	**0.72** (↓) *
Kininogen-1	KNG	P08934	23.51	43.2	20	1.06	1.04	**1.27** (↑) *
Plasma protease C1 inhibitor	SERPING1	Q6P734	21.73	39.3	14	**1.28** (↑) *	0.94	1.01
Protein C	PROC	Q68FY8	4.17	22.1	2	**1.33** (↑) *	1.35	1.10
Protein C1r (Fragment)	C1R	D4A1T6	8.03	19.4	4	1.15	**1.40** (↑) *	1.25
Protein RGD1564515 (Fragment)	RGD1564515	F1LYQ4	11.49	49.6	7	**0.66** (↓) *	**0.66** (↓) *	1.13
Serine protease inhibitor A3K	SERPINA3K	P05545	30.76	62.5	30	0.84	0.89	**1.53** (↑) *
Serine protease inhibitor A3M (Fragment)	SERPINA3M	F1LR92	18.03	44.3	14	1.10	1.06	**1.45** (↑)
Serine protease inhibitor A3N	SERPINA3N	P09006	29.27	62.4	26	**1.37** (↑) *	**0.67** (↓) *	0.90
T-kininogen 1	KNG1	P01048	8.01	63.3	33	1.38	**0.45** (↓) *	0.64
T-kininogen 2	KNG2	P08932	45.83	65.4	34	**1.95** (↑) *	**0.56** (↓) *	**0.68** (↓) *
Uncharacterized protein	Ighm	F1LN61	35.49	71.1	41	**0.69** (↓) *	**0.60** (↓) *	0.81
Uncharacterized protein	Ighm	F1LM30	25.71	50	20	**0.67** (↓) *	**0.47** (↓) *	**0.49** (↓) *
Uncharacterized protein (Fragment)	Ighm	F1LPQ6	18.67	63.4	17	**0.75** (↓) *	**0.49** (↓) *	0.84

The proteins that show *p* < 0.05 are indicated in bold and marked with “*”.

## Supplementary Materials

Supplementary materials can be found at www.mdpi.com/1422-0067/17/10/1644/s1.
